# Different methods of calculating ankle-brachial index in mid-elderly men and women: the Brazilian Longitudinal Study of Adult Health (ELSA-Brasil)

**DOI:** 10.1590/1414-431X20165734

**Published:** 2016-11-24

**Authors:** M. Miname, I.M. Bensenor, P.A. Lotufo

**Affiliations:** 1 Centro de Pesquisa Clínica e Epidemiológica do Hospital Universitário, Universidade de São Paulo, São Paulo, SP, Brasil; 2 Instituto do Coração, Universidade de São Paulo, São Paulo, SP, Brasil; 3 Faculdade de Medicina, Universidade de São Paulo, São Paulo, SP, Brasil

**Keywords:** Peripheral artery disease, Risk factors, Atherosclerosis, Cardiovascular risk, Primary care

## Abstract

The ankle-brachial index (ABI) is a marker of subclinical atherosclerosis related to health-adverse outcomes. ABI is inexpensive compared to other indexes, such as coronary calcium score and determination of carotid artery intima-media thickness (IMT). Our objective was to identify how the ABI can be applied to primary care. Three different methods of calculating the ABI were compared among 13,921 men and women aged 35 to 74 years who were free of cardiovascular diseases and enrolled in the Brazilian Longitudinal Study of Adult Health (ELSA-Brasil). The ABI ratio had the same denominator for the three categories created (the highest value for arm systolic blood pressure), and the numerator was based on the four readings for leg systolic blood pressure: the highest (ABI-HIGH), the mean (ABI-MEAN), and the lowest (ABI-LOW). The cut-off for analysis was ABI<1.0. All determinations of blood pressure were done with an oscillometric device. The prevalence of ABI<1% was 0.5, 0.9, and 2.7 for the categories HIGH, MEAN and LOW, respectively. All methods were associated with a high burden of cardiovascular risk factors. The association with IMT was stronger for ABI-HIGH than for the other categories. The proportion of participants with a 10-year Framingham Risk Score of coronary heart disease >20% without the inclusion of ABI<1.0 was 4.9%. For ABI-HIGH, ABI-MEAN and ABI-LOW, the increase in percentage points was 0.3, 0.7, and 2.3%, respectively, and the relative increment was 6.1, 14.3, and 46.9%. In conclusion, all methods were acceptable, but ABI-LOW was more suitable for prevention purposes.

## Introduction

The ankle-brachial index (ABI) is the ratio of systolic blood pressure measured at the ankle - at the pedis dorsalis or posterior tibial artery - divided by the systolic blood pressure measured in the arm at the brachial artery. A lower ABI value is associated with multiple occlusions between the aorta and the distal limb arteries (1,2). The ABI has been presented as an important tool for diagnosing peripheral artery disease (PAD), although its role as a screening test for PAD is controversial (3). Furthermore, an abnormal ABI was able to re-classify the Framingham Risk Score, in women more than in men (4). For the purpose of PAD diagnosis, ABI has been calculated by assessing the highest value of the ankle blood pressure to estimate the maximum perfusion pressure in the limb (ABI-HIGH) (5). In contrast, Schroder et al. (6) showed that using the lowest ankle blood pressure for the numerator of the ABI (ABI-LOW) increases the sensitivity for PAD assessed by arterial duplex sonogram with no loss of specificity. The results of the AtheroGene study revealed that the use of ABI-HIGH underestimated the risk for cardiovascular events (7). The Multi-Ethnic Study of Atherosclerosis presented a different approach at baseline, adding a new ABI calculation by using the average of the arm blood pressure measurements (ABI-MEAN). The odds ratios of ABI-HIGH for association with subclinical atherosclerosis were the greatest compared to the other methods (8). However, the sensitivity of ABI-LOW was better than ABI-HIGH (8). Furthermore, a scientific statement from the American Heart Association did consider that the lowest of the limb pressures is a more reliable method to risk-stratify individuals (ABI-LOW) (9). To verify this new statement, the researchers conducting The Genetic Determinants of Peripheral Arterial Disease study (The GenePAD) on 1,413 patients who underwent an elective coronary angiogram for coronary heart disease (CHD) evaluation compared the results of the 5-year follow-up for all-causes mortality and cardiovascular mortality; the hazards ratios were higher for ABI-LOW than ABI-HIGH, without the loss of combined sensitivity and specificity when applying the ABI-LOW (10). The Heinz Nixdorf RECALL (risk factors, evaluation of coronary calcium and lifestyle) study, with a 5-year follow-up, revealed a higher association between ABI-LOW and PAD than ABI-HIGH and PAD (11). A reappraisal of three National Health and Nutrition Examination Surveys that measured ABI revealed that different methods can alter substantially the number of people eligible for secondary prevention (12).

One hypothesis to be tested is how incremental ABI-MEAN or ABI-LOW will perform for overall cardiovascular risk evaluation compared to the traditional calculation (ABI-HIGH). The Brazilian Longitudinal Study of Adult Health presents a unique opportunity to test this association among 15,105 apparently healthy adult men and women (12–17).

## Material and Methods

ELSA-Brasil is a cohort study described elsewhere in detail (12–16). Briefly, it follows 15,105 voluntary civil servants participants aged 35–74 years living in 6 Brazilian cities (Belo Horizonte, Porto Alegre, Rio de Janeiro, Salvador, São Paulo, and Vitoria). They were enrolled for follow-up addressing cardiovascular and diabetes as primary outcomes. History of previous cardiovascular disease and smoking status was self-reported. Anthropometric and physiological parameters were measured by trained nurses using standard equipment and techniques. Diagnoses of hypertension (15), diabetes (16), and dyslipidemia (17) were obtained using standardized methods described elsewhere.

### ABI measurement

Ankle and brachial pressure were measured by a trained and supervised team of nurses using an automatic blood pressure monitor (Omron model HEM-705 CP, Japan). We did not use a mercury sphygmomanometer for all participants because of legal environmental restrictions on mercury disposal in some Brazilian states. The systolic pressure at the brachial artery was measured three times with the subject in a supine position, at 2-min intervals. Next, systolic blood pressure at the posterior tibial artery was measured three times in both legs at 2-min intervals. The first blood pressure measurements in the arm and legs were not included in the calculation of mean values.

### Lower ABI cut-off definition

We used the ABI inferior cut-off of 1.0 instead the classical ABI <0.9, and compared it to the values between 1.00 and 1.39. The ABI <1.0 cut-off was selected after consulting the results of a systematic review of 25 studies with 4,186 subjects, which compared oscillometric and Doppler measurements. This meta-analysis concluded that oscillometry is a reliable and practical method, but it recommended changing the ABI cut-off to <1.0 in contrast with the classic ABI <0.9 when oscillometric devices are used (18). The Heinz Nixdorf RECALL study, which applied the oscillometric method, used an ABI cut-off of <1.0 (11).

### ABI calculation methods

We created three different ways to determine ABI based on the numerator by computing the four values for blood pressure in the limbs. ABI-HIGH was the highest value, ABI-MEAN was the average, and ABI-LOW was the lowest of these same four values for blood pressure in the limbs. The denominator was the same for all ABI, i.e., the higher of the two supine systolic blood pressure readings at the brachial artery.

### Intima-media thickness (IMT) measurement

The intima-media thicknesses of 1-cm portions of the distal left and right common carotid artery far walls were obtained by ultrasonography and automatically calculated using MIA software (Medical Imaging Applications, USA) over three cardiac cycles. More detailed information about IMT measurement in the ELSA-Brasil study can be found elsewhere (19,20). We used the maximum IMT value obtained on either side of the carotid arteries. For the purpose of this analysis, we chose two categorical variables. One was IMT values below and above the 75th percentile, and the other category was defined by values greater or less than 1 mm of thickness observed at least in one common carotid artery.

### Sampling and statistical analysis

Of the 15,105 participants, we included 14,894 (98.6%) who had the three blood pressure readings taken by an automatic device at each of the tibial and right brachial arteries. We excluded 973 participants who reported coronary heart disease, heart failure, or stroke, totaling 13,921 people. Additionally, we excluded persons with ABI >1.4, regardless of computation method, and the total varied for each category as described in Table 1.

**Table:**
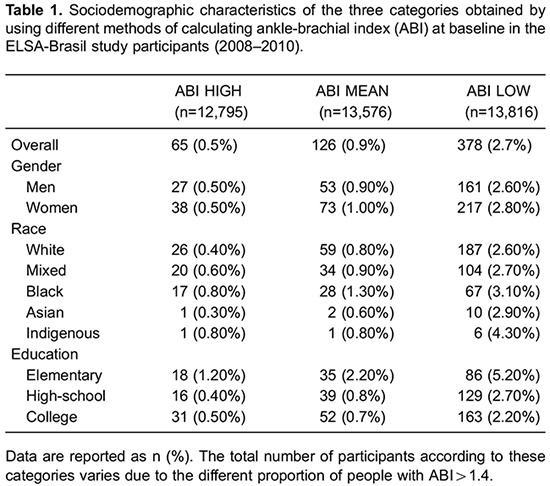

Continuous variables are reported as means±SD, while categorical variables are reported as frequency and percent. The *t*-test was used to compare means between parametric distribution variables. The chi-square test was used for comparison between categorical variables. To identify the association between ABI and cardiovascular risk factors, we applied binary logistic regression analysis using ABI as the dependent variable, and risk factors as independent variables adjusted for age, gender, ethnicity, and research site.

To compare ABI with a marker for subclinical atherosclerosis, we used the two different categories of IMT: greater than the 75th percentile, and thickness greater than 1 mm. We applied logistic regression using IMT as the dependent variable and ABI (categorical) adjusted for age (continuous), gender, ethnicity, and smoking status, diagnosis of hypertension, diabetes, dyslipidemia, and research site. The application of the Framingham Risk Score provided the 10-year risk of hard CHD (<10%, 10–20%, and >20%) (21).

Sensitivity, specificity, and likelihood ratios for the three ABI methods were tested against the two IMT categories as the gold standard. Sensitivity was the ratio between the number of participants with ABI <1.0 and undesirable IMT by the total number of people with ABI <1.0. Specificity was the ratio between the number of participants with normal ABI and low IMT values by the total number of people with normal ABI. The positive likelihood ratios were defined as sensitivity divided by 1-specificity, and the negative likelihood ratios were obtained by dividing 1-sensitivity by specificity.

## Results

Table 1 shows that the prevalence of ABI <1.0 calculated as ABI-LOW was five-fold the frequency obtained when ABI-HIGH was used, and three-fold the frequency when ABI-MEAN was applied. This pattern was the same for gender and race. Regardless of the method of computation, no differences of ABI <1.0 between men and women were observed, but black participants had a higher frequency of low ABI compared to whites. ABI-LOW increased with age more than the other two categories. The most important finding is that the method of calculating ABI presents a different pattern of increase according to age strata, as described in Supplementary Figure S1. The prevalence of ABI <1.0 using ABI-LOW was highly significant, as it was in participants under 65 years of age.

Table 2 shows that participants with ABI <1.0 compared to participants with normal ABI, regardless of the method of calculation, were older, more likely to be smokers, and to be diagnosed with hypertension, diabetes, or dyslipidemia. A high frequency of these risk factors was more visible when the category ABI-HIGH was used, compared to ABI-MEAN and ABI-LOW. Applying logistic regression (Table 3) allowed us to identify an association with age for all three methods and to confirm that there was no difference in ABI <1.0 for gender. The presence of ABI <1.0 was more frequent among blacks compared to whites only when ABI-HIGH was used. Current smoking was the most relevant risk factor for ABI <1.0. Dyslipidemia did not reach statistical significance for association with altered ABI. Hypertension was not associated with ABI <1.0 for the ABI-HIGH calculation but it was associated with ABI-MEAN and ABI-LOW.

**Table:**
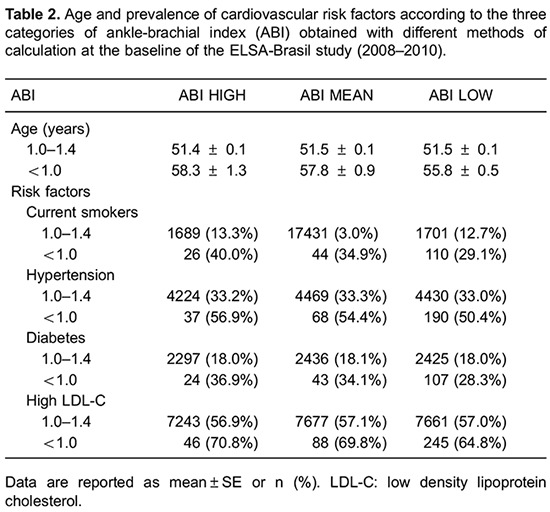

**Table:**
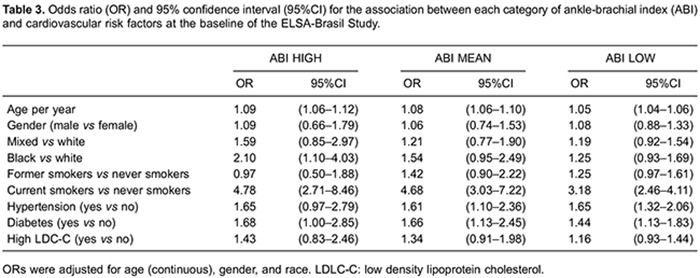

To test the strength of association between the different ABI categories and a subclinical marker of atherosclerosis such as the common carotid artery IMT, we applied logistic regression and found that ABI <1 doubles the association with an altered IMT, but the odds ratios were higher for ABI-HIGH compared to ABI-MEAN and ABI-LOW (see Supplementary Figure S2).

Another approach described in Table 4 was to compare the sensitivity and specificity of any ABI category considering IMT as the gold standard. Regardless of the calculation method, ABI <1.0 has a low sensitivity and a high specificity for the reference test using either the 75th percentile of IMT or IMT >1 mm. However, sensitivity and the positive likelihood ratio were superior when ABI-LOW was used compared to ABI-HIGH.

**Table:**
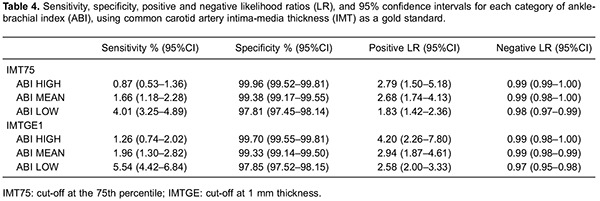

One important point is that each of these methods of calculation can alter the proportion of people at high risk for CHD. Table 5 shows that when applying the 10-year CHD risk of the Framingham Risk Score at baseline to the ELSA-Brasil participants, the method of ABI calculation matters. The proportion of participants with a 10-year risk of CHD >20% without the inclusion of ABI <1.0 was 4.9%. For ABI-HIGH, ABI-MEAN and ABI-LOW, the increase in percentage points was 0.3, 0.7, and 2.3%, respectively, and the relative increment was 6.1, 14.3, and 46.9%.

**Table:**
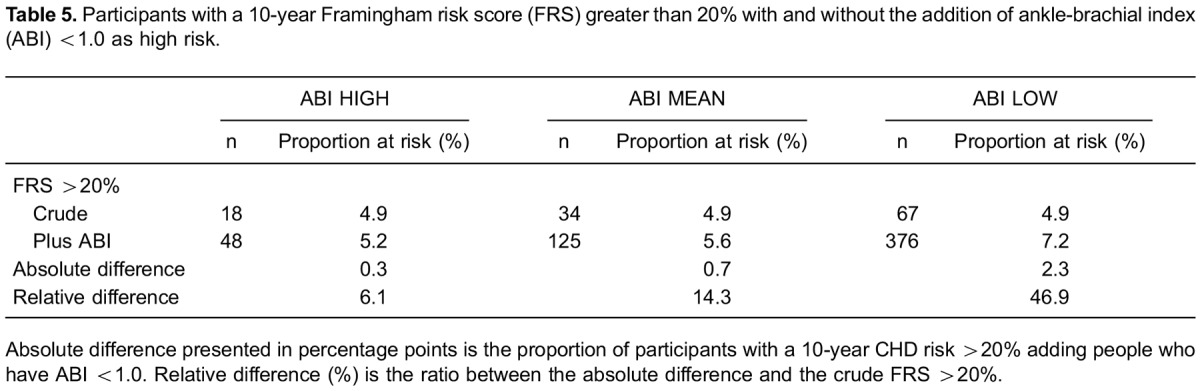

We applied a sensitivity analysis to identify a modification effect of previous disorders, as cancer and thyroid dysfunction, but the results did not change substantially.

## Discussion

In this large Brazilian cohort study of apparently healthy adults, after excluding participants with ABI>1.4, the prevalence of ABI<1.0 differed according to the method of computation used. Consequently, the prevalence of peripheral artery disease can vary five-fold when comparing ABI-LOW and ABI-HIGH in the same sample. The association with cardiovascular risk factors and with a marker for subclinical atherosclerosis presented a higher magnitude when ABI-HIGH was used, compared to ABI-MEAN and ABI-LOW. All methods showed low sensitivity and high specificity. A substantial increase in the frequency of participants classified as having high risk for CHD was observed only when ABI-LOW was applied.

The aim of our study was to identify a marker for cardiovascular prevention, rather than determine an index for measuring the prevalence of peripheral artery disease. One reason is that our cohort (median age= 51 years) is relatively young compared to others, and the frequency of PAD is high in the elderly. As described in Supplementary Figure S1, PAD prevalence rates vary widely depending on the method adopted, and only the follow-up of our participants will allow us to confirm which method of calculation will be the best for predicting PAD incidence in the sample.

One criticism of our manuscript can be related to the use of the oscillometric method for measuring blood pressure in the legs instead of Doppler. ELSA-Brasil is not the first large longitudinal study adopting this approach. The Heinz Nixdorf RECALL is also applying this method with the same 1.0 ABI cut-off (11). For the purpose of a primary care setting and use in a cardiovascular prevention program, the oscillometric method is simple, less time consuming, and cheap (22–25). Moreover, the variability of this device was lower compared to Doppler when applied in multicenter studies (25).

Our findings were relatively similar to data obtained by MESA considering IMT as a marker for subclinical atherosclerosis. ABI-HIGH had the highest odds ratio for association with IMT. In contrast, ABI-LOW had the highest sensitivity for IMT alterations in both the ELSA-Brasil and MESA studies.

There was no difference in the ABI by gender in our study, regardless of the method of calculation. These results were similar to the MESA study (8), but different from those of the Heinz-Nixdorf RECALL study, where the frequency of ABI<0.1 was higher among women (11). The association with risk factors in ELSA-Brasil was greater for smoking habit, diabetes, and high LDL-c, with the highest odds ratios using ABI-HIGH. Similar results were described in the GenePAD and MESA studies. In the Heinz-Nixdorf RECALL study, this pattern occurred only for diabetes, and in MESA for all risk factors including hypertension, which in ELSA-Brasil was only related to ABI-MEAN and ABI-LOW, but not to ABI-HIGH.

Reclassification of people with high risk of CHD by adding ABI to the Framingham Risk Score is useful (4). In the ELSA-Brasil cohort, all methods of calculation altered the number of persons at risk, but the use of ABI-LOW increased the proportion of participants with a 10-year CHD risk >20%. This finding is in accordance with the GenePad results and with Hiatt’s proposal to include ABI as a marker for CHD risk (26). Although ABI-LOW shows the weakest association with subclinical atherosclerosis, its higher sensitivity allows the addition of more people classified as high-risk for CHD events.

The limitations of our study are the cross-sectional design and the use of an oscillometric device. However, we addressed this question in an apparently healthy sample of middle age adults with a racial diversity that differs from other studies. The 32% increase of people reclassified as high-risk by the Framingham Risk Score applying ABI-LOW compared to ABI-HIGH can contribute to improving cardiovascular prevention programs. We are not advocating that one particular method of calculation is better than another, but instead showing that the method of calculation matters according to the aim of different studies and proposals.

The use of the lowest measure of leg systolic blood pressure for ABI calculation improves sensitivity and reclassifies more individuals as high-risk according to Framingham Risk Score, increasing the number of people eligible for secondary cardiovascular prevention. However, the choice of which ABI method to use depends on the hypothesis to be tested.

## Supplementary material

Click here to view [pdf].

## References

[1] Carter SA (1968). Indirect systolic pressures and pulse waves in arterial occlusive
diseases of the lower extremities. Circulation.

[2] Yao ST, Hobbs JT, Irvine WT (1969). Ankle systolic pressure measurements in arterial disease affecting the
lower extremities. Br J Surg.

[3] Lin JS, Olson CM, Johnson ES, Whitlock EP (2013). The ankle-brachial index for peripheral artery disease screening and
cardiovascular disease prediction among asymptomatic adults: a systematic evidence
review for the U.S. Preventive Services Task Force. Ann Intern Med.

[4] Fowkes FG, Murray GD, Butcher I, Folsom AR, Hirsch AT, Couper DJ (2014). Development and validation of an ankle brachial index risk model for
the prediction of cardiovascular events. Eur J Prev Cardiol.

[5] Hirsch AT, Haskal ZJ, Hertzer NR, Bakal CW, Creager MA, Halperin JL (2006). ACC/AHA 2005 Practice guidelines for the management of patients with
peripheral arterial disease (lower extremity, renal, mesenteric, and abdominal
aortic): a collaborative report from the American Association for Vascular
Surgery/Society for Vascular Surgery, Society for Cardiovascular Angiography and
Interventions, Society for Vascular Medicine and Biology, Society of
Interventional Radiology, and the ACC/AHA Task Force on Practice Guidelines
(Writing Committee to Develop Guidelines for the Management of Patients With
Peripheral Arterial Disease): endorsed by the American Association of
Cardiovascular and Pulmonary Rehabilitation; National Heart, Lung, and Blood
Institute; Society for Vascular Nursing; TransAtlantic Inter-Society Consensus;
and Vascular Disease Foundation. Circulation.

[6] Schroder F, Diehm N, Kareem S, Ames M, Pira A, Zwettler U (2006). A modified calculation of ankle-brachial pressure index is far more
sensitive in the detection of peripheral arterial disease. J Vasc Surg.

[7] Espinola-Klein C, Rupprecht HJ, Bickel C, Lackner K, Savvidis S, Messow CM (2008). Different calculations of ankle-brachial index and their impact on
cardiovascular risk prediction. Circulation.

[8] Allison MA, Aboyans V, Granston T, McDermott MM, Kamineni A, Ni H (2010). The relevance of different methods of calculating the ankle-brachial
index: the multi-ethnic study of atherosclerosis. Am J Epidemiol.

[9] Aboyans V, Criqui MH, Abraham P, Allison MA, Creager MA, Diehm C (2012). Measurement and interpretation of the ankle-brachial index: a
scientific statement from the American Heart Association. Circulation.

[10] Nead KT, Cooke JP, Olin JW, Leeper NJ (2013). Alternative ankle-brachial index method identifies additional at-risk
individuals. J Am Coll Cardiol.

[11] Kroger K, Lehmann N, Moebus S, Schmermund A, Stang A, Kalsch H (2013). Impact of atherosclerotic risk factors on different
ankle-brachial-index criteria - results of the Heinz Nixdorf RECALL
study. Vasa.

[12] Reed JF, Eid S, Edris B, Sumner AD (2009). Prevalence of peripheral artery disease varies significantly depending
upon the method of calculating ankle brachial index. Eur J Cardiovasc Prev Rehabil.

[B13] Bensenor IM, Griep RH, Pinto KA, Faria CP, Felisbino-Mendes M, Caetano EI (2013). [Routines of organization of clinical tests and interviews in the
ELSA-Brasil investigation center]. Rev Saúde Pública.

[B14] Lotufo PA (2013). [Setting up the longitudinal study for adult health
(ELSA-Brasil]. Rev Saúde Pública.

[15] Chor D, Pinho Ribeiro AL, Sa Carvalho M, Duncan BB, Andrade Lotufo P, Araujo Nobre A (2015). Prevalence, awareness, treatment and influence of socioeconomic
variables on control of high blood pressure: Results of the ELSA-Brasil
study. PLoS One.

[16] Schmidt MI, Hoffmann JF, de Fatima Sander DM, Lotufo PA, Griep RH, Bensenor IM (2014). High prevalence of diabetes and intermediate hyperglycemia - The
Brazilian Longitudinal Study of Adult Health (ELSA-Brasil). Diabetol Metab Syndr.

[17] Lotufo PA, Santos RD, Figueiredo RM, Pereira AC, Mill JG, Alvim SM (2016). Prevalence, awareness, treatment, and control of high low-density
lipoprotein cholesterol in Brazil: Baseline of the Brazilian Longitudinal Study of
Adult Health (ELSA-Brasil). J Clin Lipidol.

[18] Verberk WJ, Kollias A, Stergiou GS (2012). Automated oscillometric determination of the ankle-brachial index: a
systematic review and meta-analysis. Hypertens Res.

[19] Santos IS, Bittencourt MS, Oliveira IR, Souza AG, Meireles DP, Rundek T (2014). Carotid intima-media thickness value distributions in the Brazilian
Longitudinal Study of Adult Health (ELSA-Brasil). Atherosclerosis.

[20] Santos IS, Alencar AP, Rundek T, Goulart AC, Barreto SM, Pereira AC (2015). Low impact of traditional risk factors on carotid intima-media
thickness: The ELSA-Brasil cohort. Arterioscler Thromb Vasc Biol.

[21] Wilson PW, D'Agostino RB, Levy D, Belanger AM, Silbershatz H, Kannel WB (1998). Prediction of coronary heart disease using risk factor
categories. Circulation.

[22] Benchimol A, Bernard V, Pillois X, Hong NT, Benchimol D, Bonnet J (2004). Validation of a new method of detecting peripheral artery disease by
determination of ankle-brachial index using an automatic blood pressure
device. Angiology.

[B23] Nelson MR, Quinn S, Winzenberg TM, Howes F, Shiel L, Reid CM (2012). Ankle-Brachial Index determination and peripheral arterial disease
diagnosis by an oscillometric blood pressure device in primary care: validation
and diagnostic accuracy study. BMJ Open.

[B24] Clairotte C, Retout S, Potier L, Roussel R, Escoubet B (2009). Automated ankle-brachial pressure index measurement by clinical staff
for peripheral arterial disease diagnosis in nondiabetic and diabetic
patients. Diabetes Care.

[25] Vierron E, Halimi JM, Tichet J, Balkau B, Cogneau J, Giraudeau B (2009). Center effect on ankle-brachial index measurement when using the
reference method (Doppler and manometer): results from a large cohort
study. Am J Hypertens.

[26] Hiatt WR (2013). Should an alternate ABI definition be adopted to evaluate
risk?. J Am Coll Cardiol.

